# Factors affecting university image among graduate alumni: A case study of Qatar university

**DOI:** 10.1016/j.heliyon.2022.e09668

**Published:** 2022-06-08

**Authors:** Khalifa A. Haza, Abdel-Salam G. Abdel-Salam, Mohammad D. Mollazehi, Radwa Ismail Mohamed, Mahmood A. Ahmed, Rusol A. Al-Tameemi, Ahmed Bensaid, Chithira Johnson, Michael H. Romanowski

**Affiliations:** a Student Affairs Office, Qatar University, Doha, Qatar; b Student Experience Department, Qatar University, Doha, Qatar; c Institutional Survey Research, Institutional Research and Analytic Department, Qatar University, Qatar; d Educational Research Center, College of Education, Qatar University, Doha, Qatar; e Mathematics, Statistics and Physics, College of Arts and Sciences, Qatar University, Doha, Qatar

**Keywords:** University image, Postgraduate students, Reputation

## Abstract

Various factors influence the students' perception of universities and university image. This study explores five factors affecting university image among Graduate Alumni. Surveys, administered to 597 graduate alumni were assessed to determine Graduate Alumni perspectives toward their university. Findings revealed that the key factors that impacted graduate alumni affecting the university's image and reputation were gender, nationality, level of study, and the ability of the institution to equip graduates with certain specific skills. Based on these findings, the university should consider further examining these areas to provide a more in-depth understanding of how these factors work to shape graduate students' perspectives of the university and develop ways to address areas that need to be developed and improved.

## 1 Introduction

Graduate schools are an essential element in the education sector. Enrolling in a graduate school is a significant investment for an individual and requires evaluating both professional and personal long-term goals (Blagg and Blom, 2018; Tran, 2017). There are several benefits to enrolling in graduate education. Career development, enrichment of prospects, acquisition of in-depth knowledge, and increased productivity are a few reasons (Fernandez et al., 2019). The education system worldwide has diversified immensely, leading to increased graduate school enrollment. This is also the case in Qatar, where newer institutions, degree programs, and increasing student enrollment have been observed. Every institution aims to create an environment that fosters quality learning, propagates knowledge, and increases satisfaction and a positive image, thereby maintaining a competitive edge over others (Shanka et al., 2006).

Graduate schools play an essential role in shaping individual attributes and competencies among their alumni. Some of the competencies gained include management and soft skills, research and analytical skills, teamwork and leadership, problem-solving and decision-making, and initiative and enterprise skills. Under the management and soft skills, enrolling in a graduate course equips individuals with the attributes required to enhance individual productivity and career growth in their respective workplaces (Wickam et al., 2015). Additionally, graduate courses expose students to research work, as they must work on a thesis or a dissertation, which helps sharpen research and analytical skills (Munns et al., 2014). Graduate school curricula emphasize teamwork and leadership, making it essential to perform exceedingly well in future workplaces (Shook and Keup, 2012). Problem-solving and decision-making are vital in graduate school since they facilitate career growth. At the same time, initiative and enterprise skills sharpen the capability of an individual to be more creative in the workplace by introducing new ideas (Baird and Parayitam, 2019). Consequently, graduate students' key competencies and attributes enable them to be more open-minded and effective communicators and encourage them to value diversity in their future workplace (Kumar, 2009). Based on these competencies, pursuing graduate education is essential because it facilitates career growth for an individual.

Various factors influence the students' perception of universities and university image. Alumni tend to evaluate their universities based on the effectiveness of the knowledge acquired and the impact it is bound to have on their career growth. They are likely to review the institute's performance in facilitating a learning program for each competency mentioned above (Mbawuni and Nimako, 2015). Prospective graduate students consider a wide range of factors to determine whether the learning environment in a higher education institute is conducive and worthy of investment. In addition, employers can judge a university by determining how well its graduates engage in teamwork and exhibit leadership skills (Polziehn, 2011). Employers may quickly determine whether graduate alumni, namely alumni that graduated from a graduate school, associated with a specific graduate school can introduce new ideas to the organization and how effective their problem-solving skills are. Reviews from both alumni students and employers play an essential role in shaping the image and reputation of an institution (Drydakis, 2016; Mcadoo, 2010).

Consequently, it is necessary for all institutions enrolling graduate students to identify strategies to utilize available resources in equipping learners with all the key competencies sought out in the job market. Subsequently, a positive image and reputation will increase the competitive edge of an institution in the education sector. In light of this, the present study examines the key factors that impacted graduate alumni's views of QU's image and investigate the relationship between these factors and university image.

## 2 Context of the study

Considering that Qatar University (QU) is a high-rising national university in the Gulf region, it is important to understand how graduates perceive this institution and the factors that affect its image. This research aims to identify the opinions of graduate alumni about QU to measure and assess the university's image.

A limited number of research studies have focused on Qatar University's image (Alhaza et al., 2021). For instance, El-Kassem (2020) affirms that QU must enhance its image to remain competitive in today's globalization era. She suggests that QU could achieve a positive, attractive image by adopting nationally approved student curricula, hiring highly qualified professors, increasing its media visibility, and committing to achieving academic excellence in the long term. She intimates that only when QU implements these suggestions will the university excel.

Sellami et al. (2017) contend that today's need for STEM professionals has increased. They indicate that STEM courses would be most befitting to develop Qatar's economy, based on its heavy reliance on petroleum and natural gas. Therefore, it is proposed that Gulf Cooperation Council (GCC) countries (the United Arab Emirates, Saudi Arabia, Qatar, Oman, Kuwait, and Bahrain) improve their course delivery to attract students willing to undertake STEM courses. Hence, QU can only produce skilled students in STEM to strengthen the economy only if it improves its course delivery quality.

Finally, Shurair and Pokharel (2019) compare quality services in higher education institutions to the business environment, which is essential to maintaining client loyalty, building a positive brand image, and attracting clients. Therefore, for QU to remain viable in the highly competitive higher education industry, it must improve its service delivery quality. This will eventually lead Qatar to attract a pool of highly talented students who will be loyal to the institution until they complete their studies. It is suggested that if QU improves its quality of education services provided to students, the institution could even attract learners from international markets, given that today the rate of globalization has increased exponentially.

Several studies examined the point of view of parents, high-school students, and stakeholders toward the QU image (El-Kassem et al., 2018; El-Kassem, 2020; Shurair and Pokharel, 2019). This study adds to the research on QU's image by examining the factors that university image among graduate school alumni. Therefore, this research is significant in filling the research gap that examines graduate school students' perceived ideas of the university image in the GCC. However, the current study will evaluate how the skills acquired by graduate students can influence the university image, as the existing literature does not assess this association. Since the findings identify the key factors that impacted graduate alumni and their view of the university, the research can be helpful for universities throughout the Gulf Cooperation Council countries (GCC) with comparable cultures concerned about its graduate students' perspective of the university and its programs. Therefore, the proposed research will fill the corresponding gaps using factual, scientific evidence. It will also contribute to the existing literature concerning the local and regional higher education sector by introducing the graduate alumni perception using a Graduate Alumni Survey.

## 3 Theoretical background

Marketing researchers introduced the relationship marketing theory as one of the best alternatives to foster customer loyalty and build a marketing network (Ehigie and Taylor, 2009). According to Morgan and Hunt (1994, p .22), relationship marketing theory is defined as “all marketing activities directed towards establishing, developing and maintaining successful relational exchanges”. In education, relationship marketing theory can be described as building an enduring relationship network with the university's graduates. This study uses five factors that affect university image as a framework to examine graduate school alumni’ perception of Qatar University's image. These factors have been identified within the research as management and soft skills (Fahrner and Schüttoff, 2020; Lamberti et al., 2021; Osipov et al., 2021), research and analytical skills (Alkharusi et al., 2019; Marcus, 2009), teamwork and leadership (Finch et al., 2013; Heslop and Nadeau, 2010), initiative and enterprise (Iglesias-Sánchez et al., 2019; Watts et al., 2010), and problem-solving and decision-making (Alcaide-Pulido et al., 2017; Finch et al., 2013). These factors influence the quality of education that affects the Qatar university's image, as it would in any other university.

## 4 Literature review

### 4.1 University image

University image is “the result of an aggregate process using students' mental perceptions of their reality, based on the evaluation of various university attributes through the expression of their feelings, ideas, beliefs, impressions, and real-life experiences at the university” (Manzoor et al., 2021, p. 222). Musselin (2018) argues that university image is vital for higher education institutions since competition is increasing, but new forms of competition have emerged. Specifically, competition is about quality, and higher education institutions are employed in a quality competition. Lafuente-Ruiz-de-Sabando et al. (2018) suggest that higher education institutions are increasingly investing resources to achieve good perceptions among their stakeholders. The university's image and reputation are established on how well its graduates have gained key competencies vital for the workplace. It is an essential factor in enhancing student satisfaction.

Most students are influenced by university affective, cognitive, and overall image. The affective component of the image is entirely linked with the person's emotional reactions. The image might also include responses deemed bad or good, negative or positive, judgmental or evaluative, likable and desirable (Agapito et al., 2013). Azoury et al. (2014) contend that the cognitive element of the image in a Middle Eastern context works as a predecessor of the affective component. Both components improve the university's overall image, which considerably influences students' overall satisfaction with their university (Azoury et al., 2014).

Several research scholars have highlighted some factors that substantially influence the creation of a university image in its graduates' minds. These factors include rules and regulations, progressive faculty, diverse coursework, and research and development (El-Kassem, 2020; Hussain et al., 2020; Stephenson and Yerger, 2015). Other scholars have stated that academic networks, social networks, reputation, organizational history, and employability positively influence the image of a university (Aghaz et al., 2015). Furthermore, Chandra et al. (2019) addressed university image as an institutional factor of precedence to sustain its position in the competitive market. Personal factors about students can also significantly influence university images, such as nationality and ethnicity (Azoury et al., 2014; Wilkins and Huisman, 2015), gender (Wilkins and Huisman, 2015), level of study (Alves and Raposo, 2010; Azoury et al., 2014) and the ability of the institution to equip graduates (Gupta, 2009; Majid et al., 2012; Mitchell et al., 2010).

### 4.2 Alumni at the workplace and organizations

**Figure 1:**
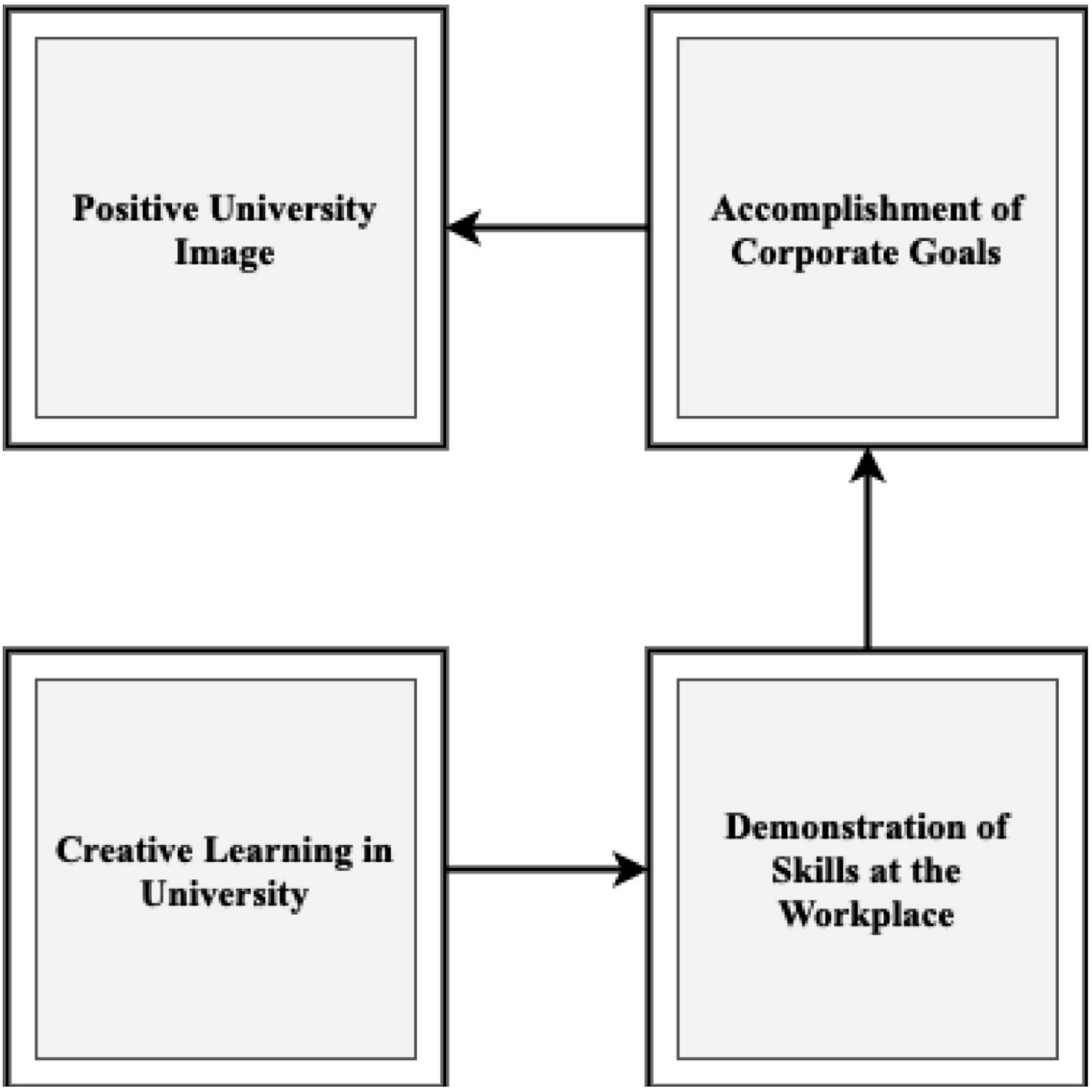
Influence of university image.

Creative learning in the university predicts the individual's behavior at the workplace. For instance, Beghetto and Karwowski (2018) have revealed that when an individual learns creative skills in university, it is likely for the respective individual to demonstrate such skills in the workplace. As a result, creative learning in university substantially influences individuals' demonstration of skills at the workplace. Costa and Pelissari (2017) have portrayed that when a specific alumnus accomplishes corporate goals at the workplace, the organization can recruit more talented alumni from the same university. In addition, Wibowo et al. (2020) concluded that an organization could accomplish its corporate goals when individuals actively demonstrate their workplace skills. Sanyal and Hisam (2018) considered that teamwork-based workplace skill is essential for enhancing the overall employees' performance that accomplishes corporate goals. They also stated that effective organizational culture, trust climate, leadership style, and reward mechanism add value to goal accomplishment. From this perspective, the university image is a substantial outcome of creative learning, demonstrating skills, and achieving corporate goals; such process of university image creation is represented in Figure 1.

### 4.3 Factors of university image among alumni

Five factors affecting university image among Graduate Alumni will be explored. Management and soft skills, research and analytical skills, teamwork and leadership skills, problem-solving and decision-making, and initiative and enterprise.

#### 4.3.1 Management and soft skills

Management consists of four sub-factors, namely, planning, organizing, leading, and controlling the ability of the individuals. Day-by-day jobs require additional management skills from graduate alumni (Plice and Reinig, 2007). On the other hand, soft skills are described as personality traits that contribute to creating a successful workforce (Al-Hashimi et al., 2020). Fahrner and Schüttoff (2020) have argued that individuals with a high personality trait score will increase productivity. From this perspective, it becomes clear that advanced managerial skills will enable alumni to perform well in the workplace. So, one may claim that when alumni perform at the workplace, they represent their educational institutes by their performance, that will upsurge their university image.

#### 4.3.2 Research and analytical skills

Research and analytical skills can be described as cognitive knowledge, which an individual learns from his educational institute during his academic course (Hamilton et al., 2016). In this context, analytical skills include forecasting, researching, and problem-solving. In this regard, Galante et al. (2018) demonstrated that when students possess appropriate research and analytic skills, they will think critically about their ongoing work. As a result, they will demonstrate their best by applying analytical knowledge (Changwong et al., 2018). This, in turn, will enhance the university's compelling image (Alkharusi et al., 2019). Moreover, research quality and effectiveness can impact the regional growth and development of the country, leading to the creation of a positive image for the university (Cunningham et al., 2019; Valero and Van Reenen, 2019). Therefore, the graduate alumni's research and analytical skills in their educational institutes will positively influence the university's image in the workplace.

#### 4.3.3 Teamwork and leadership

Every organization struggles to attain a competitive advantage over several other organizations by implementing and selecting well-known and effective mechanisms (Al-Hashimi et al., 2020). From this perspective, the effective use of internal human resources in universities, such as graduate alumni, positively predicts organizational success (Haage et al., 2021). Currently, organizations follow effective leadership and teamwork that will ultimately bring positive market outcomes. The skills that graduate alumni reported gaining by researching through their university experience include working independently, working as a leader, and speaking proficiently (Hamilton et al., 2016). By performing those skills on the work front by the graduate alumni, the university's image will be honed and shaped.

#### 4.3.4 Problem solving and decision-making

Problem-solving skills encompass an individual's critical thinking and reasoning skills. Individuals with effective problem-solving skills will ultimately have strong communication skills by collaborating, cooperating, listening, speaking up, and asking questions (Erozkan, 2013). Research indicated that when alumni have strong communication skills, it will ultimately enable them to produce positive outcomes for the organization (Fahrner and Schüttoff, 2020). The effective application of these skills at the workplace will ultimately attract employers to recruit employees possessing similar skills, most likely from the same educational institution (Frensch and Funke, 2014). Along with other variables, Lavi et al. (2021) found problem-solving and decision-making skills to be decisive factors for forming a positive image for the university. Therefore, one can deduce that problem-solving skills at the workplace ultimately influence the university's image of alumni.

#### 4.3.5 Initiative and enterprise

Initiative and enterprise refer to individuals having done their required work without question. It ultimately means creative thoughts and problem-solving skills (Fahrner and Schüttoff, 2020). They encompass the ability to identify new ideas and opportunities for one's business and practices. In the context of university image, initiative and enterprise are referred to as introducing and exploring new opportunities to attract more students. It also includes introducing new ideas by which students can be motivated (Viatkina, 2014).

#### 4.3.6 Alumni characteristics

Personal characteristics play a vital role in developing skills, which are essential in increasing productivity in the workplace (Pedro et al., 2018). For instance, research portrayed that leadership skills at the workplace substantially depend on alumni's characteristics: a comprehensive strategic plan, a digital-first media strategy, and the alumni gateway to the institution (Kotschevar et al., 2018; Whittington and Garst, 2018). Students are encouraged to equip themselves with constructive skills to exhibit higher productivity.

Dillon (2017) demonstrated that alumni characteristics such as age, income, and engagement in university events increase their ability to demonstrate higher productivity at the workplace. Consequently, it has a significant influence on the university's image. In this regard, Holmes (2009) has revealed that students who attend campus events have a 17% higher attachment with the university than those absent from the on-campus events. It implies that such attachment enables alumni to learn more skills to groom their talents. Consequently, the job market can predict the university's image by measuring the alumni's performance (Salem and Mobarak, 2019).

Similarly, Christian (2018) found that demographic characteristics form a substantial source for employers to predict the university's image. He commented that personal traits such as being female, having a middle-income job, being of the age 25–33 years, and academic satisfaction from the offered courses are more associated with creating the university image. Additionally, Langdon et al. (2011) mentioned a much more significant projected growth of STEM-related employment than in other fields. In this regard, it can be stated that the personal characteristics of the alumni, such as age, income, gender, nationality, STEM, non-STEM, and GPA, might be significant predictors of university image.

## 5 Methodology

### 5.1 Research questions and hypotheses

This survey was conducted in the academic year 2018–2019. All documents required by Qatar University's Institutional Review Board (QU-IRB) were submitted for review. The data set was obtained from the university's student satisfaction and senior student survey that received informed consent from participants. Based on the source of data and the researchers' inability to identify participants, QU-IRB determined that the study was deemed a quality improvement project or an audit classified as non-human subject and did not require IRB review and approval (QU-IRB 002-NR/20).

The subsequent section will provide a detailed description of the Graduate Alumni survey used to gather the required information. Based on the literature and the discussion of the factors that impact graduate alumni's perception of the university image, this research formulated the multiple hypotheses below:

$H_{1a}:$ There is a relationship between image and graduate alumni acquiring self-management and soft skills.

$H_{2a}:$ There is a relationship between image and graduate alumni with research and analytical skills.

$H_{3a}:$ There is a relationship between image and graduate alumni acquiring teamwork and leadership skills.

$H_{4a}:$ There is a relationship between image and graduate alumni with problem-solving and decision-making skills.

$H_{5a}:\;$ There is a relationship between image and graduate alumni with initiative and enterprise skills.

$H_{6a}:$ There is a relationship between image and graduate alumni characteristics.

$H_{7a}:$ There is a relationship between image and the effect of field of study on the workplace.

### 5.2 Instrument development

The Graduate Alumni Survey was designed electronically using the Qualtrics software consisting of 41 items based mainly on responses of Graduate school alumni to questions that assess the knowledge, skills, and abilities earned during their study in QU. In addition to collecting the basic demographic information about the respondents, the survey included 31 Likert-scale items starting at “Not Applicable,” scaling up to “Strongly Agree.” The 5-point Likert-scale questions are divided into three parts related to developing technical and soft skills, curriculum quality, and workplace performance. Four single-answered multiple-choice items and six open-ended questions are also added to the survey allowing respondents to provide more information about their major and study experience. One single-answered item “Would you recommend your family members or friends to study at QU?” included an open-ended follow-up question to specify the reason if the response was “No.” All instructions and items in the survey were written in Arabic then translated to English. Alumni university emails are used to send the Arabic and English versions of the survey (see AppendixesA, B & C). To reduce non-response, multiple dimensions of the survey were administered, namely a personable yet professional introduction, interesting survey content, short survey length, clear and concise wording, practical and appealing incentives, placement of multiple follow-up calls or email reminders to non-responders and taking into account the time, day or season.

The Graduate Alumni Survey content validity was confirmed by reviewing and revising the previous version by the Institutional Survey Research Section at the university and cooperating with representatives of colleges and academic and administrative units dealing with graduate students. The review was conducted following the performance indicators required for their strategic plans and services needed to be evaluated. Then, the final review was completed, and the Institutional Survey Review Committee approved the survey.

To assess the convergent validity and reliability of the instrument, the researchers used the average variance extracted (AVE), standardized factor loadings and Cronbach's alpha. While the discriminant validity was measured using Fornell Larcker's criterion (FLC) and the Heterotrait-Monotrait ratio of correlations (HTMT).

### 5.3 Data collection

For the present investigation, the Graduate Alumni Survey was designed in determining the following:

- Satisfaction of academic knowledge, skills, and abilities acquired during their study.
- Perception of the university image.
- Effect of a field of study on the workplace.

The study received an exemption from the Institutional Review Board approval since the research involved the study of data existing institutional data, which is publicly available, and the information was recorded by the researchers in such a manner that subjects cannot be identified.

The Survey was sent in April 2019 to 597 graduate alumni from the cohorts 2016–2017 and 2017–2018 via email. Several email reminders were sent to increase the response rate, and phone calls were made to non-respondents. An email was sent to colleges coordinators requesting them to contact their alumni and encourage them to participate in the survey. The final response rate was 43.5%, with a margin of sampling error of ±4%.

### 5.4 Sample characteristics

The survey included background information about graduate alumni to gather some socio-demographic data. A total of 260 that adequately completed the survey were tabulated and used for further analysis. In general, 75% of the student body in the university is female (Qatar University, 2018). The female response to the survey was 60.0%. It is worth mentioning that the university hosts students from 90 countries. However, nationals make up a higher proportion of registered students (Qatar University, 2018). Nevertheless, the majority of responses are from non-nationals, as can be seen in Table 1. Another observation is that the proportion of employed individuals is higher among the sample (see Table 2).

**Table 1:** Graduate Alumni Characteristics.

Gender	
Female	60.0% (156)
Male	40.0% (104)
Nationality	
National	30.4% (79)
Non-national	69.6% (181)
Education Level	
Diploma	11.2% (29)
Masters	80.4% (209)
Ph.D.	8.5% (22)
Major Classification	
STEM	41.9% (109)
Non-STEM	58.1% (151)
Employment	
Employed	84.9% (219)
Unemployed	15.1% (39)
GPA^a^	*M* = 3.51
	*SD* = .52

a Continuous variable.

**Table 2:** Measurement model results.

Constructs	Code	Items	SL^a^	SE^b^	t	α	CR^c^	AVE^d^	VIF^e^
Self-Management and Soft Skills									
	SMSK1	Choose my career path	0.843	0.022	37.519	0.724	0.845	0.645	2.456
	SMSK3	Work with interdisciplinary fields	0.785	0.036	21.865				
	SMSK4	Engage in intellectual activities	0.780	0.041	18.959				
Research and Analytical Skills									
	RAS1	Synthesize and analyze data	0.788	0.040	19.549	0.699	0.831	0.621	1.890
	RAS2	Design and execute research	0.754	0.050	14.930				
	RAS3	Identify and utilize appropriate research methodological tools	0.820	0.033	24.631				
Teamwork and leadership									
	TL1	Work effectively as a team member	0.785	0.042	18.635	0.756	0.860	0.672	2.402
	TL2	Acquire effective Leadership skills	0.865	0.020	43.776				
	TL3	Work effectively with people from diverse backgrounds	0.808	0.043	18.724				
Problem Solving and Decision-making									
	PSD2	Demonstrate socially responsible decision-making	0.748	0.048	15.437	0.814	0.877	0.641	3.019
	PSD3	Apply IT effectively in decision-making	0.786	0.029	26.764				
	PSD4	Integrate the functional areas of knowledge in decision-making	0.855	0.020	43.751				
	PSD5	Consider global issues in decision-making	0.810	0.030	26.842				
Initiative and Enterprise									
	IE1	Think critically	0.707	0.052	13.515	0.794	0.866	0.619	3.664
	IE3	Understand discipline related concepts and apply them in real life situations	0.828	0.020	40.934				
	IE4	Analyze problems and strategies and propose solutions	0.814	0.034	23.931				
	IE5	Connect with the community by tackling practical challenges	0.794	0.031	25.558				
Effect of field of study on the workplace									
	W1	My current job is related to my major field of studies	0.798	0.057	14.086	0.702	0.856	0.749	1.584
	W2	My major helped me in my performance at the workplace	0.928	0.018	51.007				
Image									
	IM1	The competency of QU postgraduate alumni are comparable to those of graduates from other universities	0.795	0.031	25.379	0.842	0.887	0.612	
	IM2	QU's reputation is comparable to other universities in terms of quality of its alumni	0.773	0.038	20.216				
	IM4	Quality of QU alumni is continuously improving	0.709	0.046	15.549				
	IM5	QU academic programs are "high quality" programs	0.839	0.023	36.460				
	IM6	QU offers academic programs suitable to the labor market in the State of Qatar and the region	0.790	0.025	31.044				

a Standardized loading.

b Standard error.

c Composite Reliability.

d Average Variance Extracted.

e Variance Inflation Factor.

The university offers 55 graduate degrees in nine fields (Arts and Sciences, Business and Economics, Education, Engineering, Law, Medicine, Pharmacy, Sharia and Islamic Studies, and Health Sciences). The graduate programs awarded by QU encompass 27 Master's degrees, 19 doctoral degrees, four diplomas, four certificates, and a PharmD (Qatar University, 2018). Most respondents indicated that they obtained a master's degree, while the remainder were roughly equally distributed among other levels of education (Diploma and Ph.D.). Non-STEM majors slightly outnumbered STEM majors (58.1% vs. 41.9%) among the different specializations. Of 260 respondents, 109 individuals reported specializations classified as STEM following the mapping of Turner and Brass's (2014) report.

### 5.5 Survey results

The first section of the survey consisted of items targeting students' skills and knowledge during their studies. Of the respondents in the sample, 55.8% strongly agreed that the academic experience allowed them to think critically, 54.3% synthesize and analyze data, 59.9% design and execute research, 53.7% find and apply information, 54.3% work effectively within a team, and 55.4% identify the appropriate methodological tools. However, 26.4% strongly agreed that the university could help them acquire writing funding proposals. The second section investigated the general perception of the university. The majority of respondents, 62.7%, strongly agreed that university reflects the national identity.

Moreover, 51.6% of the respondents strongly agreed that the university offers high-quality programs, and 52.4% thought that the quality of its alumni is comparable to other universities. The last section in the survey consisted of three questions investigating how the skills earned from the university helped in the workplace. In addition, 55.3% of the graduate alumni strongly agree that their major helped their work performance. In comparison, 25.7% strongly agreed that their graduate degree contributed to their promotion at work, and 32.4% somewhat agreed with this statement.

### 5.6 Analysis and results

#### 5.6.1 Structural equation model (SEM) approach

The proposed model and hypotheses in this study were investigated via SEM, using the Smart-PLS software. Ullman and Bentler (2003) contend that a latent variable model should reduce measurement errors resulting in bias. Since this study's factors were measured using a questionnaire, the latent variable model is appropriate.

##### 5.6.1.1 Reliability

This research examined the standardized factor loadings to assess all items' reliability. Any standardized factor loading greater than 0.7 is suitable (Hair et al., 2011). This study used two-tailed p-values to confirm the appropriateness of factor loadings.

For each construct, the standardized factor loadings are presented in Table Two. The table demonstrates at the item level, convergent validity exceeded 0.7 and was significant at the level of 0.001. All items with a standardized factor loading of less than 0.7 were removed during path analysis. As Hair et al. (2011) suggest, the study evaluated construct reliability using Cronbach's alpha and composite reliability to determine construct reliability greater than 0.7. See Table Two for information on construct reliability scores.

#### 5.6.2 Convergent validity

The average variance extracted (AVE) was used to assess the convergent validity. Hair et al. (2011) suggested the convergent validity should be larger than 0.5. Table Two illustrates that all constructs have an acceptable convergent validity.

##### 5.6.2.1 Discriminant validity

Fornell Larcker's criterion (FLC) and the Heterotrait-Monotrait ratio of correlations (HTMT) were used to measure discriminant validity where the square root of AVE of each factor must be greater than the absolute value of their correlation coefficients (off-diagonal). Table Three demonstrates the square root of AVE on the diagonal and the absolute value of the correlation coefficients for the factors. AVE's square root was greater than the correlation coefficients (off-diagonal) for all factors. The study used HTMT to test the discriminant validity. The HTMT value should be less than 0.85 to be acceptable (Henseler et al., 2014). According to Table 3, the maximum HTMT value (0.85) signifies that discriminant validity is established for this model (see Table 4).

**Table 3:** Discriminant validity results.

	A	B	C	D	E	F	G
(A) Image	**0.783**	0.791	0.759	0.596	0.583	0.731	0.632
(B) Initiative and Enterprise	0.657	**0.787**	0.766	0.587	0.845	0.849	0.842
(C) Problem Solving and Decision-making	0.641	0.633	**0.824**	0.535	0.516	0.693	0.674
(D) Relation between Education and Job	0.493	0.468	0.430	**0.886**	0.424	0.703	0.421
(E) Research and Analytical Skills	0.463	0.635	0.402	0.323	**0.788**	0.728	0.669
(F) Self-Management and Soft Skills	0.578	0.643	0.541	0.528	0.526	**0.803**	0.835
(G) Teamwork and leadership	0.512	0.690	0.542	0.328	0.503	0.619	**0.820**

**Table 4:** *Structural model results*.

	Path coefficient	Boot S.E	t-value (bootstrap)	p-value	95% Confidence interval
***Alumni Characteristics***					
Gender (G)					
Male	0.085	0.042	2.063	0.039	(0.006, 0.170)
Female	*Ref*				
Nationality (N)					
Nationals	0.104	0.048	2.201	0.028	(0.011, 0.201)
Non-nationals	*Ref*				
Major Classification (M)					
STEM	-0.010	0.056	0.144	0.886	(-0.120, 0.099)
Non-STEM	*Ref*				
Employment Status (ES)					
Employed	-0.015	0.046	0.322	0.748	(-0.105, 0.076)
Unemployed	*Ref*				
Education Level (EL)					
PhD	-0.110	0.050	2.235	0.025	(-0.214, -0.019)
Master	-0.173	0.047	3.700	0.000	(-0.271, -0.085)
Diploma	*Ref*				
GPA	-0.035	0.036	0.866	0.387	(-0.099, 0.040)
***Factors***					
Initiative & Enterprise Skills (IES)	0.233	0.079	3.013	0.003	(0.088, 0.396)
Research and Analytical Skills (RAS)	0.057	0.061	0.908	0.364	(-0.061, 0.178)
Problem Solving and Decision-making Skills (PSDS)	0.311	0.065	4.784	0.000	(0.176, 0.430)
Teamwork and leadership Skills (TLS)	0.034	0.069	0.415	0.678	(-0.111, 0.159)
Self-Management & Soft Skills (SMSK)	0.142	0.073	1.981	0.048	(0.009, 0.296)
Effect of the field of study on the workplace (EFSW)	0.119	0.051	2.327	0.020	(0.016, 0.213)

#### 5.6.3 Structural model assessment

This research assesses the structural model and testing hypothesis (Sarstedt et al., 2017). The Variance Inflation Factor (VIF) value was examined to test collinearity before the structural relationship results analysis.

The study reported that the structural model had minimal collinearity. Indeed, after evaluating all potential sets of constructs for collinearity, the obtained VIF values were less than 5. Furthermore, $R^{2}$, the coefficient of determination measures the whole effect size and variance, which measures the model's predictive accuracy. Instead, the model's predictive relevance, $Q^{2}$, measures the path model's quality and in this study, the $R^{2}$ statistic was 58.5%, which indicates that the structural model's overall effect size is moderate. The $Q^{2}$ statistic was reported as 33.9%, higher than the threshold of zero. This suggests that the path model's predictive relevance is adequate.

#### 5.6.4 Model fit

Pavlov et al. (2020) suggest that calculating the standard root-mean-square residual (SRMR) provides a goodness-of-fit measurement that can be used as an index to validate models. Based on this, zero values in the SRMR indicate a perfect fit since the model contains an absolute measure of fit, although the model deems a value of less than 0.08 as generally a good fit (Hu and Bentler, 1999). Because the value obtained was 0.059 for this study, the path model is fitted with the empirical data.

#### 5.6.5 Hypotheses testing

The derived hypotheses are exemplified by the size and significance levels of the path coefficients. Using a PLS-procedure that included a resampling bootstrapping process consisting of 10,000 bootstrap samples and 260 bootstrap cases, the significance levels of the path coefficients were found. Table Four shows the path coefficients, standard errors, significance level, t-values, and the bootstrap confidence intervals, 95%. This means that the three alumni characteristic variables and four factors are significant.

The findings indicate that the highest alumni characteristic effect was for the education level ($\beta_{Master}$ = -0.173, and $\beta_{PhD}$ = -0.110), followed by nationality ($\beta_{Nationals}$ = 0.104) and gender ($\beta_{Male}$ = 0.085). Moreover, in terms of factors, the effect of problem-solving and decision-making skills (PSDS) ($\beta_{PSDS}$ = 0.311), initiative and enterprise skills (IES) ($\beta_{IES}$ = 0.233), self-management and soft skills ($\beta_{SMSK}$ = 0.142), and effect of field of study on the workplace (EFSW) ($\beta_{EFSW}$ = 0.119) were statistically significant.

## 6 Discussion

The study revealed that the key factors that impacted graduate alumni affecting QU's image were gender, nationality, level of study, and the ability of the institution to equip graduates with certain specific skills. Regarding gender, these findings indicated that male graduate alumni reported a more positive view of the university's image than their female counterparts. This finding aligns with research that reported that gender influenced university image (Al Hazaa et al., 2021; Clemes et al., 2013). However, this finding also breaks from other research findings that indicate that gender did not significantly influence graduates' image of the university (Aghaz et al., 2015; Sultan and Wong, 2019). Polat (2011) reported that students' perception of the organizational image of a university predicts the academic success of its students. Based on this, it might be inferred that the better image among the male graduate alumni in QU than the females could be attributed to the academic level of performance.

Another outcome is a variation in perception based on nationality or between Qatari and non-Qatari graduate alumni. The Qatari graduate alumni have a better image of the University than the non-Qatari graduate alumni. Previous studies have reported that nationality influenced university image (Al Hazaa et al., 2021; Wilkins and Huisman, 2011, 2015). For example, Kazoleas et al. (2001) report that studies often compared the university they attended to other universities within their home state based on the quality of programs, faculty commitment, and services provided to students. In this study, one could argue that non-national alumni tend to compare the resources and experiences in their home universities with what they experience at QU. Lack of resources aids in developing a negative image, especially when their home institutions offer much better experiences (Xie, 2008).

Furthermore, Karuppan and Barari (2010) report that perceived discrimination has a strong, negative impact on the educational experience. Perceived discrimination which is nearly exclusively negative increased stress and identity conflict, decreased academic contentment, and creates psychological and sociocultural adjustment problems. When students experience discrimination, their image of the university is influenced (Leong and Ward, 2000). QU has specific situations where nationals and non-nationals are treated differently based on nationality, which would shade non-nationals' experiences at QU. That is not the situation for the national graduate alumni who are less likely to be victims of discrimination and negative experiences.

One of the key findings from the investigation is that the university's image is less favorable as the level of study increases. There is a better image among the diploma graduate alumni when compared to the master's graduates. Also, there is a better image among the masters when compared to the Ph.D. graduate alumni. This supports previous research. Alves and Raposo (2010) reported a variation in the satisfaction and loyalty of students under the various programs in a learning institution. This variation in satisfaction is what creates the image of the learning institution. Diploma students in most universities are not accorded the needed attention compared to Ph.D. students. The variation makes a distinction in the perception and image of the university. Azoury et al. (2014), in their investigation, support this outcome of having a less positive image as one moves up the program ladder. They posit that the satisfaction of the students is what creates the difference. For example, Ph.D. learners have access to most resources restricted to graduate students. Some libraries are set aside for undergraduate students in several universities, giving them a different satisfaction and experience from the postgraduate students.

These findings indicate that the main factor affecting QU's image is the institutional ability to produce graduates with efficiency in particular sets of skills. These skills may include problem-solving skills, initiative and enterprise skills, workplace performance skills, or even self-management skills. There are previous studies that support this finding. Studies report that students' qualifications, in this sense, refer to the qualities and skills obtained from the university significantly contribute to the definition of quality of education, which influences the university's image (Aghaz et al., 2015; Akareem and Hossain, 2012). Hodgman (2018) found that employers expect higher education institutions to provide graduates at all levels with the knowledge and skills necessary to be successful in the workplace. These skills include speaking (Stevens, 2005) and writing skills (Goldschmidt, 2005). Also, technological skills are among the skills needed by workers (Ghannadian, 2013), as are intercultural skills (Hansen and Hansen, 2015). When graduates demonstrate competency in these skills, employers develop a positive view of the graduate's university (Pang et al., 2019). As stated above, all elements of the quality of education and curriculum or method of teaching ought to contain such skill-building elements. Graduate alumni are concerned about these skills because graduate institutions and potential employers often look for a fine blend of competencies and soft skills (Majid et al., 2012).

In some past studies, alumni characteristics are not usually treated as an antecedent factor for university image but instead as an outcome of university characteristics or a moderation variable. Rajassekharan et al. (2020) investigate the alumni's loyalty based on the observed image of their universities. The results indicated that three university image variables determine student loyalty: reputation, usability and shared values. Schlesinger et al. (2017), similarly, also tested the roles of brand image, trust, satisfaction, and shared values on the loyalty levels of alumni. The results revealed that alumni loyalty is impacted directly by satisfaction, shared values, and trust, and a mediation path impacts university image. Schlesinger et al. (2021) also found university brand image to be a key driver of positive word of mouth for alumni.

Finally, graduate institutions and potential employers perceive that professional and technical skills alone are not enough to attain organizational objectives and goals (Mitchell et al., 2010) since they are expected to be involved in divergent leadership and decision-making activities. As found in this research, soft skills are essential to employers and can influence the university's image. Hodges and Burchell (2003) depicted that more than 80% of the top ten skills that potential employers look for included soft skills such as willingness and ability to learn, cooperation and teamwork, passion and energy, interpersonal communication, and critical thinking and problem-solving skills. Therefore, the alumni are primarily concerned about acquiring these soft skills because it affects them directly regarding getting admittance into postgraduate programs and attaining employment at prominent organizations (Wats and Wats, 2009). The better the delivery of soft skills by Qatar University, the better its image.

## 7 Limitations and future research

There are several limitations to the study. One is that of the sample size. The sample size used in the investigation consists of 260 participants. The power of the study is in the ability to detect an effect in situations where there is none for detection (Faber and Fonseca, 2014). Not having a large sample size in the investigation resulted in type two errors, which skewed the outcomes. The action results declined the power of the investigation. Another limitation is that the study only targeted graduate alumni. That entails students that graduated from diploma, master's, and Ph.D. programs from the university. The issue is a limitation because the outcomes will be based on these groups and the study might include a wide range of views in the research, such as the faculty workers. Also, the study narrowly defines university image as equipping students with skills and knowledge required for the workplace. Many other factors are involved in a university's image that is not addressed and would be worthwhile for future studies.

Also, future studies should embark on the study of factors beyond acquired skills that affect the university image. Some of these factors may include cost and availability of financial aid packages, level of parents' education, campus location, availability of certain assemblages, and other non-academic related factors. This would help shed light on other extrinsic non-academic factors that affect the university's perception and image. There is a need for future studies to focus on the university's image from the point of view of undergraduates and graduate students. The undergraduates and graduate students are in direct connection with the university. They can provide information about their experiences in the university, which will create the needed image. Lastly, additional studies are needed to equally focus on the university's image from the perspective of the facility members and the staff. The working staff builds on the rapport of the university. They have information on the benefits they receive, contributing to the institution's image. The viewpoint of the recruiters should be included in future studies as they are in close connection with the facility's functioning. They can narrate if the university's effectiveness is fit to build on a good image.

## 8 Conclusion

Lafuente-Ruiz-de-Sabando et al. (2018) contend that higher education institutions invest significant resources to achieve recommendatory perception among their stakeholders. University image is a complex issue with many factors influencing stakeholders' perspectives. However, the image that universities hope to project does not always align with alumni perceptions, demonstrating that image management is not lacking challenges. Thus, universities need to assess the university image of students (Duarte et al., 2010) and analyze image dimensions to identify the critical area for improvement (Manzoor et al., 2021, p. 222).

This study aimed to identify the key factors that impacted graduate alumni's views of QU's image and determine if there was a relationship between these factors and university image. More specifically, the findings from this study indicate that several factors influence graduate students' image of QU. These factors were gender, nationality, level of study, and the ability of the institution to equip graduates with certain specific skills.

Based on these findings, there are several recommendations for universities. To ensure that students are satisfied with the university's service, which influences image, Green et al. (2015) suggest that universities should identify factors on a "satisfaction importance grid" to help determine what areas the university can improve. Factors considered highly important for alumni and found to be low satisfaction are logical targets for improvement. For example, teaching factors usually relate highly and greatly influence students' view of the university's image. This is a logical target to improve satisfaction (Green et al., 2015). In addition, based on the findings that the university's image is less favorable as the level of study increases, universities should target postgraduates to improve satisfaction.

Nguyen and Leblanc 2001 argue that alumni can influence a university's image by endorsing the image and reputation often by encouraging word of mouth (Helgesen and Nesset, 2007). Therefore, institutions need to contemplate institutional and personal factors (Chahal and Devi, 2013). The university should consider further examining these areas to provide a more in-depth understanding of how these factors work to shape graduate students' perspectives of the university and develop ways to address areas that need to be developed and improved. In addition, these findings can assist the university in shaping the uniqueness of the institution and aid in marketing decisions. Addressing these factors will influence how stakeholders view the university and improve learning and the overall university experience for graduate students.

## Declarations

### Author contribution statement

Khalifa A. Haza: Conceived and designed the experiments; Wrote the paper.

Abdel-Salam G. Abdel-Salam: Conceived and designed the experiments; Analyzed and interpreted the data; Wrote the paper.

Mohammad D. Mollazehi; Radwa Ismail Mohamed: Performed the experiments; Analyzed and interpreted the data.

Mahmood A. Ahmed: Analyzed and interpreted the data.

Rusol A. Al-Tameemi: Conceived and designed the experiments; Analyzed and interpreted the data.

Ahmed Bensaid; Michael H. Romanowski: Analyzed and interpreted the data; Wrote the paper.

Chithira Johnson: Conceived and designed the experiments; Contributed reagents, materials, analysis tools or data.

### Funding statement

This research did not receive any specific grant from funding agencies in the public, commercial, or not-for-profit sectors.

### Data availability statement

The authors do not have permission to share data.

### Declaration of interests statement

The authors declare no conflict of interest.

### Additional information

No additional information is available for this paper.

## Figures and Tables

**Table:** 

No	Item
To what extent do you agree with acquiring academic knowledge, skills and abilities during your study at QU?	
1	Think critically
2	Work with interdisciplinary fields
3	Synthesize and analyze data
4	Design and execute research
5	Choose my career path
6	Engage in intellectual activities
7	Find and apply information
8	Work effectively as a team member
9	Identify and utilize appropriate research methodological tools
10	Understand discipline related concepts and apply them in real life situations
11	Write and present reports, articles, books, business plans, etc.
12	Write funding proposals
13	Analyze problems and strategies and propose solutions
14	Manage time effectively
15	Acquire effective Leadership skills
16	Work effectively with people from diverse backgrounds
17	Demonstrate ethics in decision-making
18	Demonstrate socially responsible decision-making
19	Apply IT effectively in decision making
20	Connect with the community by tackling practical challenges
21	Integrate the functional areas of knowledge in decision making
22	Consider global issues in decision making
To what extent do you agree with the following statements about Qatar University in general?	
23	The competency of QU postgraduate alumni is comparable to those of graduates from other universities
24	QU's reputation is comparable to other universities in terms of quality of its alumni
25	QU reflects Qatar national identity
26	Quality of QU alumni is continuously improving
27	QU academic programs are "high quality" programs
28	QU offers academic programs suitable to the labor market in the State of Qatar and the region
To what extent do you agree with the following statements?	
29	My current job is related to my major field of studies
30	My major helped me in my performance at the workplace
31	QU degree has contributed to my promotion at work

**Table:** 

No	Question
1	When did you pursue your postgraduate degree at Qatar University?
2	In which sector, are you working?
3	Please, specify the field you are working in:
4	Would you recommend your family members or friends to study at QU?

**Table:** 

No	Question
1	If you work, what is your current position?
2	What are the strengths of the major that you graduated from?
3	What are the weaknesses of the major that you graduated from?
4	What could QU do to improve the curriculum of your major that you graduated from?
5	What are the majors you would like Qatar University to have?
6	Please provide us with any additional comments.
